# Biofilm-stimulated epithelium modulates the inflammatory responses in co-cultured immune cells

**DOI:** 10.1038/s41598-019-52115-7

**Published:** 2019-10-31

**Authors:** Jason L. Brown, William Johnston, Chris Delaney, Ranjith Rajendran, John Butcher, Shaz Khan, David Bradshaw, Gordon Ramage, Shauna Culshaw

**Affiliations:** 1 Institute of Biomedical and Environmental Health Research, School of Science and Sport, University of the West of Scotland, Paisley, PA1 2BE UK; 2 Oral Sciences Research Group, Glasgow Dental School, School of Medicine, College of Medical, Veterinary and Life Sciences, University of Glasgow, Glasgow, G12 8TA UK; 3 Department of Life Sciences, School of Health and Life Sciences, Glasgow Caledonian University, Glasgow, G4 0BA UK; 4 Oral Healthcare R&D, GlaxoSmithKline Consumer Healthcare, Weybridge, KT13 0DE UK

**Keywords:** Biofilms, Mechanisms of disease

## Abstract

The gingival epithelium is a physical and immunological barrier to the microbiota of the oral cavity, which interact through soluble mediators with the immune cells that patrol the tissue at the gingival epithelium. We sought to develop a three-dimensional gingivae-biofilm interface model using a commercially available gingival epithelium to study the tissue inflammatory response to oral biofilms associated with “health”, “gingivitis” and “periodontitis”. These biofilms were developed by sequential addition of microorganisms to mimic the formation of supra- and sub-gingival plaque *in vivo*. Secondly, to mimic the interactions between gingival epithelium and immune cells *in vivo*, we integrated peripheral blood mononuclear cells and CD14^+^ monocytes into our three-dimensional model and were able to assess the inflammatory response in the immune cells cultured with and without gingival epithelium. We describe a differential inflammatory response in immune cells cultured with epithelial tissue, and more so following incubation with epithelium stimulated by “gingivitis-associated” biofilm. These results suggest that gingival epithelium-derived soluble mediators may control the inflammatory status of immune cells *in vitro*, and therefore targeting of the epithelial response may offer novel therapies. This multi-cellular interface model, both of microbial and host origin, offers a robust *in vitro* platform to investigate host-pathogens at the epithelial surface.

## Introduction

The gingival epithelium is a stratified, squamous, keratinised epithelium. This epithelium provides a physical and immunological barrier to the external environment, and through the production of soluble mediators such as cytokines, chemokines and anti-microbial peptides orchestrates the immune response to microbial challenge^1^. The gingival epithelium is confronted with the bacterial consortia on the tooth or root surface. These bacteria reside as complex multi-species biofilms, i.e., three dimensionally arranged bacterial cells attached to one another and interconnected through complex carbohydrate containing polymers^2^. DNA sequencing of the oral microbiome has led to the concept of microbial “signatures” of bacteria associated with health and disease; “health-associated” bacteria consortia include a dominance of *Streptococcus* species. “Gingivitis-associated” consortia include *Fusobacterium* and *Prevotella* species, and “periodontitis-associated” bacteria include *Porphyromonas gingivalis*, *Treponema denticola* and *Tannerella forsythia*^3,4^. The epithelial cell response to oral bacteria varies depending on biofilm composition, with commensal bacteria eliciting minimal inflammatory response and pathogenic bacteria mediating an elevated pro-inflammatory response^5–8^.

Communication between the epithelium and innate and adaptive immune cells is pivotal for rapid recognition and effective elimination of pathogens at the epithelial surface^9^. There is a complex immunological network underlying the gingival epithelium, including populations of monocytes, neutrophils and T cells^10^, and the inflammatory response of immune cells cultured with periodontal pathogens has been well characterised in previous studies. In a similar manner to biofilm-epithelial cell interactions, oral bacteria elicit differential inflammatory responses in immune cells depending on the microorganisms’ pathogenicity. Studies have described the capacity for oral pathogens such as *Fusobacterium nucleatum* and *P. gingivalis* to induce a M1 (pro-inflammatory) type response in THP-1 monocyte/macrophage cell lines, whilst oral commensals elicit a M2 (anti-inflammatory/immunomodulatory) type response^11^. Dendritic cells (DCs), monocytes and neutrophils cultured with and without gingival epithelial cells or epithelial cell spent supernatants also show unique activation signatures and/or cytokine gene‐ and protein‐expression profiles^12–15^. Moreover, oral epithelial cell spent supernatants can induce phenotypic changes in human monocyte cell lines *in vitro*, suggesting that such interactions arise through epithelium-produced soluble mediators^12,15^. To our knowledge, there are studies elsewhere that describe 3D organotypic tissue co-culture models representative of different oral diseases^16,17^, but limited evidence of models integrating immune cells with multi-layered gingival epithelium cultures and oral biofilms. In the current study we sought to develop an immune cell/gingival tissue/biofilm model aiming to recapture the cell interactions at the gingival margin, encapsulating the response of the local inflammatory infiltrate.

## Material and Methods

### Bacterial growth and standardisation

Bacterial strains used in the study were as follows; *Streptococcus mitis* NCTC 12261, *Streptococcus intermedius* DSM 20753, *Streptococcus oralis* NTCC 11427, *Fusobacterium nucleatum* ATCC 10596, *Fusobacterium nucleatum* spp. *vincentii* DSM 19507, *Actinomyces naeslundii* DSM 17233 *Veillonella dispar* NCTC 11831, *Porphyromonas gingivalis* W83, *Prevotella intermedia* DSM 20706 and *Aggregatibacter actinomycetemcomitans* ATCC 43718. *S. oralis*, *S. intermedius*, *S. mitis*, *A. actinomycetemcomitans* were grown on Columbia blood agar (CBA) for 1–2 days, then in Tryptic soy broth (TSB) (Sigma, Poole, UK) for 1–2 days, at 5% CO_2_, 37 °C. The remaining bacteria were all grown on Fastidious anaerobic agar (FAA) for 2–3 days, then *A. naeslundii* and *V. dispar* were sub-cultured into Brain Heart Infusion (BHI) broth and *F. nucleatum*, *F. nucleatum* spp. *vicentii*, *P. gingivalis* and *P. intermedia* into Schaedler anaerobic broth (Oxoid, Cambridge, UK) for a further 1–2 days, all at 37 °C in an anaerobic chamber (85% N_2_, 10% CO_2_ and 5% H_2_; Don Whitley Scientific Limited, Shipley, UK).

### Growth of multi-species oral biofilms

**Figure 1:**
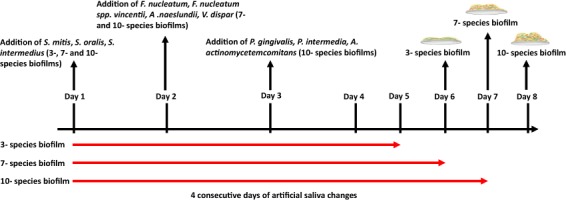
Timeline for growth of 3-, 7- and 10- species biofilms. Bacteria (1 × 10^7^ CFU/ml in artificial saliva (AS)) were sequentially added to 8 mm glass coverslips or 13 mm Thermanox coverslips for biofilm culture. Biofilms were cultured under appropriate conditions for a total of 5–7 days depending on biofilm composition, with AS changes every 24 hours for a total of 4 consecutive days (red lines). Once mature, final day supernatants and biofilms were stored at −80 °C. Biofilms were revived in AS, 24 hours prior to experimental use. Image partially created using Biorender.

Three multi-species oral biofilms representative of a “health-associated” (3- species), “gingivitis-associated” (7- species) and “periodontitis-associated” (10- species) consortium of bacteria were grown for a total of 5–7 days in artificial saliva (AS), as previously described^18,19^. Biofilms were prepared in line with a standardised protocol as previously described^20,21^, and as summarised in Fig. 1. Briefly, all bacteria were grown as above, washed three times in phosphate buffered saline (PBS), then standardised to 1 × 10^7^ CFU/ml in AS prior to sequential addition to the biofilm. All biofilms were grown statically on 8 mm diameter round glass coverslips (Thomas Scientific, New Jersey, USA) or 13 mm diameter round Nunc Thermanox plastic coverslips (Thermo Fisher, Loughborough, UK), as indicated within the text. Once formed, biofilms were frozen at −80 °C until required. It is noteworthy that biofilm composition and viability was assessed pre- and post- freezing, and the number of total and viable microorganisms remained similar (Supplementary Fig. 1).

### Quantitative analysis of multi- species biofilm composition

Biofilms were detached from coverslips by sonication at 35 kHz for 10 minutes in a sonic bath in 1 ml of PBS as previously described^22^ and the sonicate transferred to a RNase-free microcentrifuge tube. The sonicate was centrifuged (13,000 × g, 10 mins) and pellet retained for DNA extraction. Bacterial DNA was then extracted using the QIAamp DNA Mini Kit (Qiagen, Crawley, UK), according to manufacturer’s instructions. To produce bacterial standard curves, single species bacterial cultures were standardised to 1 × 10^8^ CFU/ml in sterile PBS, then DNA extracted in the same way as above. DNA from the 1 × 10^8^ CFU/ml culture was then serially diluted to 1 × 10^3^ CFU/ml in nuclease-free water, and the resulting standard curve was used to calculate the colony forming equivalent (CFE) of each bacterial species within the multi-species biofilm (Supplementary Fig. 2). Melting curve analysis was performed for all primer sets to ensure a single peak, which was indicative of primer specificity. A list of bacterial primer sequences and reference sources are listed in Table 1. Given the high degree of genetic similarity and heterogeneity between the *Streptococcus* and *Fusobacterium spp*. used in these models, one “universal” genus-specific primer set was used to detect total numbers of *Streptococcus* (*S. mitis*, *S. oralis*, *S. intermedius*) and *Fusobacterium* (*F. nucleatum* and *F. nucleatum* subsp. *vincentii*) species. To account for variation in the standard curves, the reported CFE/mL for *Streptococcus* and *Fusobacterium* is reflective of an average for all three *Streptococcus* or two *Fusobacterium* species, respectively.

**Table 1:** Primer sequences used for qPCR in this study.

Multi-species biofilm composition analysis			Reference
Bacterial spp.	Forward primer 5′−3′	Reverse primer 5′−3′	
*Streptococcus* spp.	GATACATAGCCGACCTGAG	TCCATTGCCGAAGATTCC	^65^
*A. naeslundii*	GGCTGCGATACCGTGAGG	TCTGCGATTACTAGCGACTCC	^65^
*V. dispar*	CCGTGATGGGATGGAAACTGC	CCTTCGCCACTGGTGTTCTTC	^66^
*Fusobacterium* spp.	GGATTTATTGGGCGTAAAGC	GGCATTCCTACAAATATCTACGAA	^67^
*P. gingivalis*	GCGCTCAACGTTCAGCC	CACGAATTCCGCCTGC	^68^
*P. intermedia*	CGGTCTGTTAAGCGTGTTGTG	CACCATGAATTCCGCATACG	^20^
*A. actinomycetemcomitans*	GAACCTTACCTACTCTTGACATCCGAA	TGCAGCACCTGTCTCAAAGC	^69^
**Quantitative PCR gene expression analysis of epithelial and immune cells**			
**Cytokine**	**Forward primer 5′−3′**	**Reverse primer 5′−3′**	
*IL1β*	TCCCCAGCCCTTTTGTTGA	TTAGAACCAAATGTGGCCGTG	^70^
*TNF*	CCCCAGGGACCTCTCTCTAATC	GGTTTGCTACAACATGGGCTACA	^71^
*IL6*	CAATCTGGATTCAATGAGGAGAC	CTCTGGCTTGTTCCTCACTACTC	^72^
*IL8*	CAGAGACAGCAGAGCACACAA	TTAGCACTCCTTGGCAAAAC	^73^
*IL10*	GCTGGAGGACTTTAAGGGTTACCT	CTTGATGTCTGGGTCTTGGTTCT	^71^
*GAPDH*	CAAGGCTGAGAACGGGAAG	GGTGGTGAAGACGCCAGT	^73^

### Ultrastructural changes of multispecies biofilms

The morphology and architecture of the multi-species biofilms were assessed using scanning electron microscopy (SEM), transmission electron microscopy (TEM) and confocal laser scanning microscope (CLSM) imaging. For all microscopic techniques, biofilms were grown on Nunc Thermanox plastic coverslips to fully mature, washed with PBS, then processed. For SEM, biofilms were fixed in 2% (v/v) para-formaldehyde, 2% (v/v) glutaraldehyde and 0.15 M sodium cacodylate, and 0.15% w/v Alcian Blue, pH 7.4 as described previously^23^. Following processing, the biofilms were sputter-coated with gold and viewed under a JEOL JSM-6400 scanning electron microscope at magnifications x500 and x5000. Images were assembled using Photoshop software (Adobe, San Jose, USA). For TEM, biofilms were fixed in 1.5% (v/v) glutaraldehyde and 0.1 M sodium cacodylate for 1 hour at 4 °C. Samples were then washed three times in 0.1 M sodium cacodylate buffer containing 2% sucrose (w/v) and post-fixed with 1% osmium tetroxide and 0.1 M sodium cacodylate buffer. Biofilms were then processed as previously described^24^ and visualised on JEOL1200EX TEM running at 80 kV.

For CLSM, mature biofilms were stained using the LIVE/DEAD BacLight bacterial viability kit (Thermo Fisher, Loughborough, UK) containing the fluorescent dyes, SYTO 9 and Propidium iodide (PI). These dyes were combined at a ratio of 1:1, and 1 ml was added to each biofilm and stained for 15 min in the dark at 37 °C. Biofilms were then washed with 1 ml of PBS and fixed with 2% para-formaldehyde (PFA) for 1 hour. Biofilms were again washed and mounted to glass slides for viewing under a Zeiss LSM 780 CLSM, at excitation and emission wavelengths of 488/533 nm for SYTO9 and 561/631 nm for PI, respectively. Image were taken at x 40 magnification and image stacks compiled using COMSTAT2 program (MATLAB; MA, USA).

### Human gingival epithelium co-cultured with multi-species biofilms

Human gingival epithelium (HGE) used throughout this study was purchased from Episkin (Skinethic, Lyon, France; http://www.episkin.com). HGE was composed of normal human gingival cells cultivated on an inert polycarbonate filter (0.4 µm porous membrane) at the air liquid interface for 12 days. Prior to experimental set-up, HGE was incubated with maintenance media in 12 well plates for 24 hours, 5% CO_2_ at 37 °C. Maintenance media was replaced then co-culture three-dimensional models were set up as follows.

#### HGE incubation with biofilm supernatants

**Figure 2:**
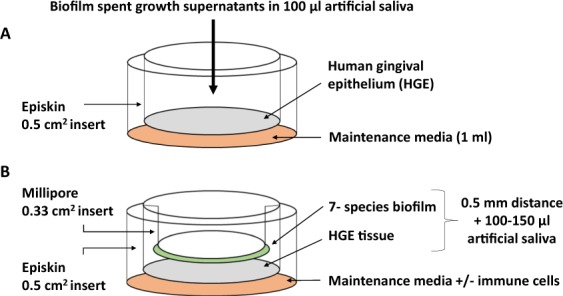
Schematic diagram depicting the three-dimensional human gingival epithelium (HGE) co-culture set-up. For biofilm supernatant co-culture experiments, HGE tissue was exposed to final day 3-, 7- and 10- species biofilm spent growth supernatants (100 µl) (**A**). For biofilm co-culture experiments, HGE tissue was co-cultured with 3-, 7- and 10- species biofilms inverted and secured to the underside of 0.33 cm^2^ inserts with sterile Vaseline (**B**). For other experiments, 0.5–1 × 10^6^ cells/ml of peripheral blood mononuclear cells or CD14^+^ monocytes were added to the maintenance media underneath the HGE tissue. All experiments were conducted for 24 hours at 37 °C, 5% CO_2_.

Final day biofilm spent growth supernatants (day 6 for 3-species biofilms, day 7 for 7-species biofilms and day 8 for 10-species biofilms; Fig. 1) were filtered through 0.2 μm membrane filters (Millipore, Livingston, UK) and 100 μl was added directly to the HGE tissue. For experiments with un-filtered supernatants, 100 μl of final day biofilm supernatants were added directly to HGE tissue. Negative controls contained 100 μl of filtered or un-filtered sterile AS for appropriate experiments (Fig. 2A).

#### HGE incubation with biofilms

Fully mature biofilms were revived in AS overnight and coverslips were inverted and attached to the underside of 0.33 cm^2^ inserts (Millipore, Livingston, UK) with sterile Vaseline, then placed carefully into 0.5 cm^2^ inserts containing the HGE tissue plus 100–150 μl of sterile AS. Negative controls contained 100–150 μl of sterile AS only (Fig. 2B). For integration of immune cells into the HGE-biofilm co-culture, immune cells were isolated and cultured in maintenance media beneath HGE for the duration of experiments.

### Isolation of peripheral blood mononuclear cells and CD14^+^ monocytes

Peripheral blood was collected from healthy volunteers (in accordance with College of Medical, Veterinary and Life Sciences, University of Glasgow ethics; project number 200160159) by venepuncture into preservative-free anticoagulant treated tubes, then layered onto an equal volume of pre-warmed Histopaque 1077 in 15 ml conical centrifuge tubes and the solution was centrifuged at 400 × g for 30 min at room temperature. Following centrifugation, the opaque layer was carefully aspirated using a Pasteur pipette and washed twice in pre-warmed Dulbecco’s-PBS (Thermo Fisher, Loughborough, UK) at 250 × g for 10 min. PBMCs were washed one final time in pre-warmed HGE maintenance media and viable cells were counted using trypan blue exclusion assay^25^. Monocytes were isolated from PBMC suspensions by positive selection using CD14 microbeads (Miltenyi Biotec, Crawley, UK). When used in accordance with manufacturer’s instructions, CD14 microbeads yields > 85% purity of CD14^+^ monocytes.

### TR146 oral keratinocytes co-cultured with multi-species biofilms

TR146 cells are an immortalised human oral keratinocyte cell line which form a non-keratinised stratified epithelium, which is a characteristic of buccal mucosa^26^. Frozen stocks of TR146 cells (1 × 10^6^ cells/ml) were seeded at 1 × 10^5^ cells/ml in 75 cm^3^ flasks (Corning, New York, USA) in Keratinocyte serum-free medium (KSFM) supplemented with 100 U/mL penicillin, 100 μg/ml streptomycin, 25 μg/ml bovine pituitary extract, 0.2 ng/mL epidermal growth factor and 0.4 mM CaCl_2_, then incubated at 37 °C, 5% CO_2_. Cells were maintained until they were 70–80% confluent at which point cells were passaged using 0.05% trypsin EDTA.

Following passaging, cells were counted before being seeded in 24-well plates at 1 × 10^5^ cells/well and incubated for 1–2 days at 37 °C, 5% CO_2_ until 70–80% confluent. Following incubation, media was removed, and cells were gently washed three times in pre-warmed PBS before experimental set up. For incubation with serially diluted suspensions of bacterial cultures, all microorganisms were standardised to their respective final CFE/ml representative of the composition of fully mature 3-, 7- and 10- species biofilms. These were serially diluted from 10^7^ to 10^4^ prior to co-culture with TR146 cells in KSFM. For experiments with final day biofilm supernatants, 250 μl of filtered or un-filtered 3-, 7- and 10- species biofilm supernatants plus 250 μl of KSFM were added directly to TR146 cells. Negative controls contained 250 μl of filtered or un-filtered sterile AS with 250 μl of KSFM. For whole intact biofilms, a similar biofilm co-culture set up was utilised as with the HGE tissue (Fig. 2B). 3-, 7- and 10- species biofilms grown on 13 mm Thermanox coverslips were inverted and attached to the underside of 0.33 cm^2^ inserts with sterile Vaseline, then placed carefully into each well containing TR146 cells and 250 μl of sterile AS and 250 μl of KSFM. Negative controls contained 250 μl of un-filtered sterile AS in 250 μl of KSFM minus biofilm.

### Validation of human gingival epithelium model; histological analysis, cell viability, gene expression and protein release

#### Histology of human gingival epithelium

HGE tissue was carefully cut from the 0.5 cm^2^ insert using a sharp probe and fixed in 10% neutral-buffered formalin prior to embedding in paraffin. A Finnesse ME + microtome (Thermo Fisher, Loughborough, UK) was used to cut 2 μm sections and tissue sections stained with haematoxylin and eosin.

#### Epithelial cell viability

To measure cell viability in HGE tissue and TR146 cells, spent cell supernatants were assayed for a lactate dehydrogenase (LDH) using the Pierce LDH cytotoxicity colorimetric assay kit, according to manufacturer’s instructions (Thermo Fisher, Loughborough, UK).

#### Gene expression in epithelial cells and immune cells

RNA from TR146 cells, HGE tissue and immune cells was extracted using the RNeasy Mini Kit (Qiagen Ltd, Crawley, UK). Following co-culture with biofilms, HGE tissue was cut from the 0.5 cm^2^ inserts and homogenised for 30 secs in RLT lysis buffer containing 0.01% (v/v) β-2-mercaptoethanol (β2ME) and 100 μl equivalent of 0.5 mm glass beads. RNA was extracted from the cell lysates according to the manufacturer’s instructions. For RNA extraction from TR146 cells or immune cells, RLT lysis buffer containing β2ME was added directly to the cell monolayer or immune cell pellets then vortexed for 30 secs, before proceeding with RNA extraction as above. RNA was quantified using a NanoDrop 1000 spectrophotometer (Thermo Fisher, Loughborough, UK). 100–250 nanograms of RNA was reverse transcribed, using either the RT^2^ First Strand cDNA synthesis kit (Qiagen, Crawley, UK) or ‘high capacity RNA‐to‐cDNA’ kit (Applied Biosystems, Foster City, CA, USA). Gene expression analysis was carried out using a custom designed RT^2^ Profiler PCR Array (Qiagen, Crawley, UK) or using SYBR GreenER based qPCR. RT^2^ Profiler PCR array contained primers specific for 12 genes of interest (*IL1α*, *IL1β*, *IL6*, *TNF*, *CSF2*, *CSF3*, *IL8*, *CXCL1*, *CXCL3*, *CXCL5*, *CCL1* and *GAPDH*), compiled based on an experimental gingivitis model^27^. Gene expression in immune cells using SYBR GreenER based qPCR was verified using the following genes of interest; *IL1β*, *IL6*, *TNF*, *IL8*, *IL10* and house-keeping gene, *GAPDH*. Primer sequences and reference sources are listed in Table 1.

#### Protein release by epithelial cells

Spent cell supernatants were harvested from HGE tissue, TR146 cells and immune cell co-cultures and assayed for interleukin-8 (IL-8) using Ready-Set-Go ELISA kits (Thermo Fisher, Loughborough, UK), according to manufacturer’s instructions.

### Statistical analysis

Graphs were compiled, and data analysed using GraphPad Prism (version 7; GraphPad Software Inc., La Jolla, USA). Normally distributed data that approximated to Gaussian distribution was analysed by two-tailed Student’s t-test to compare the means of two samples or one-way analysis of variance (ANOVA) to compare the means of more than two samples, and Tukey’s post-test was applied to the p value to account for multiple comparisons of the data. Values are presented as mean ± SEM. Statistical significance was achieved if *p < 0.05.

## Results

### Compositional analysis and ultrastructural differences in multi-species biofilms

**Figure 3:**
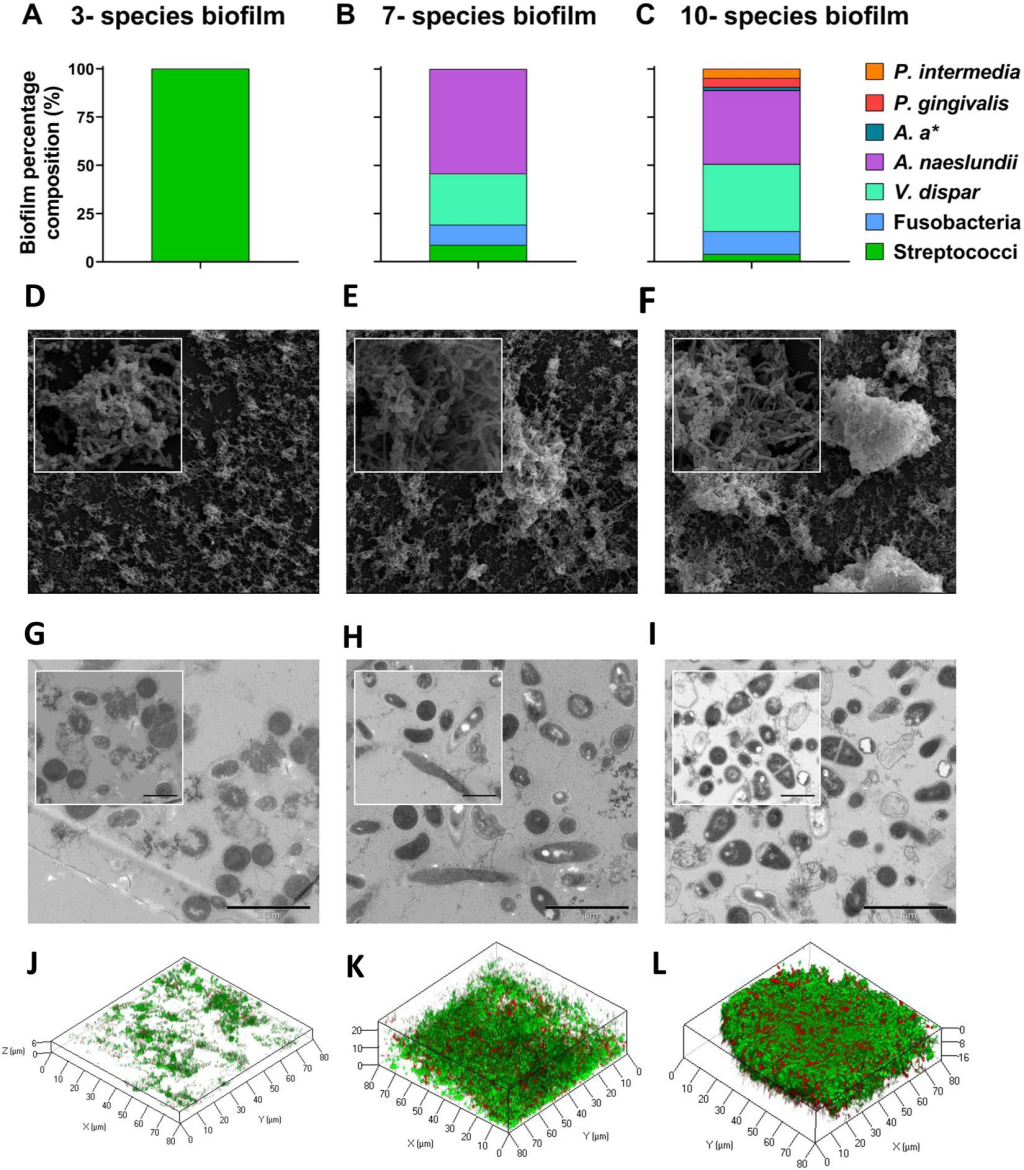
Compositional analysis and ultrastructural differences in multi-species oral biofilms. Bacterial DNA was extracted from multi- species biofilms using DNeasy Qiagen kit for quantification of each species using SYBR GreenER based quantitative PCR (**A**–**C**). Unique biofilm morphology and architecture was visualised using scanning electron microscopy ((**D**–**F**); lower magnification ×500, and higher magnification ×5000 inset) and transmission electron microscopy ((**G**–**I**); lower magnification ×15000, and higher magnification ×25000 inset). Confocal stacked images representative of biofilms stained with SYTO9 and PI to show live and dead cells as viewed using the Zeiss LSM 780 confocal laser scanning microscope. Confocal images were taken at ×40 magnification and image stacks compiled using COMSTAT2 program. Data for qPCR compositional analysis of biofilms representative of mean +/− SEM for n = 3 from two individual experiments. Abbreviations; A. a* = *Aggregatibacter actinomycetemcomitans*.

The composition of the multi-species biofilms was initially assessed using qPCR. All bacteria colonised and were represented in the final composition of the fully mature biofilms (Fig. 3A–C). Firstly, the 3 *Streptococcus* species made up 7.74 × 10^6^ CFE/ml (100%) of the 3- species biofilm. The 7- species and 10- species biofilms had similar proportions of *Streptococcus* spp. (3.26 × 10^7^ CFE/ml; 8.59% for 7- species and 1.95 × 10^7^ CFE/ml; 3.88% for 10- species) and *Fusobacterium spp*. (3.90 × 10^7^ CFE/ml; 10.46% for 7- species and 5.60 × 10^7^ CFE/ml; 11.81% for 10- species). In both 7- and 10- species biofilms, *V. dispar* and *A. naeslundii* were the most prominent microorganisms colonising the biofilm (*V. dispar*, 1.01 × 10^8^ CFE/ml; 26.58% for 7- species and 1.69 × 10^8^ CFE/ml; 34.87% for 10- species; *A. naeslundii* 2.15 × 10^8^ CFE/ml; 54.37% for 7- species and 1.90 × 10^8^ CFE/ml; 38.26% for 10- species). The 10- species biofilms also contained *P. gingivalis* (2.27 × 10^7^ CFE/ml; 4.52%), *P. intermedia* (2.54 × 10^7^ CFE/ml; 4.89%) and *A. actinomycetemcomitans* (8.19 × 10^6^ CFE/ml; 1.77%).

The architecture of the different multi-species biofilms also varied as assessed using SEM, TEM and confocal microscopic analysis. The 3- species biofilms were visibly different from the 7- species and 10- species biofilms. In SEM images, oral *Streptococcus* formed “clusters” of cocci chains across the surface of the coverslip as visible at x500 magnification. At higher magnification (x5000), extracellular matrix can be seen encapsulating the cocci chains (Fig. 3D). After TEM analysis, cocci-shaped cells are evident at x15000 and x25000 magnification (Fig. 3G). The 7- and 10- species biofilms formed a dense, complex multi-layered structure in scanning electron micrographs. At lower magnification, the broad covering of the surface with *Streptococcus* species remains visible in both biofilms, as with the 3- species biofilm, although larger micro-colonies containing rod-shaped bacteria predominate the biofilm (*Fusobacterium spp*., *A. naeslundii* and *A. actinomycetemcomitans* in the 10-species biofilm). At x5000 magnification, cell-cell adhesion between bacterial cells with different morphologies are visible, with a dense, rich extracellular matrix encapsulating the micro-colonies (Fig. 3E,F). In the TEM images for the 7- and 10- species biofilms, rod-shaped bacteria are visually interspersed between the cocci (Fig. 3H,I). Finally, the multi-species biofilms were stained with fluorescent dyes for CLSM analysis. All biofilms were predominantly stained green (~90%) suggesting that the biofilms contained predominantly viable cells. There were some regions of the 7- and 10- species that were stained red, which were indicative of dead cells, but these were sparse and largely found deeper within the biofilm structure (Fig. 3J–L).

### Histology of HGE tissue

**Figure 4:**
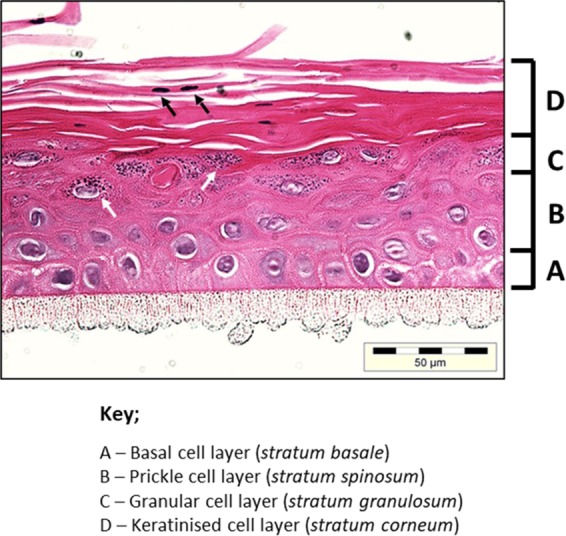
Histology of human gingival epithelium model. Haematoxylin and eosin (H&E) stained histological section of human gingival epithelium (HGE) showing the characteristic 6–8 epithelial layers of gingival tissue. The H&E stained epithelium could be further divided into characteristic cellular layers as depicted by (**A–D**) (basal cell layer, (**A**); prickle cell layer, (**B**); granular layer, (**C**); keratinised layer, (**D**)). Keratinocytes within the granular layer are identified by the presence of keratohyalin granules, shown as by the white arrows. Terminally differentiated keratinocytes (corneocytes), are identified by black arrows. Histological image representative of an unstimulated HGE tissue.

HGE used in this study formed a multi-layered keratinised epithelial tissue characteristic of gingival epithelium *in vivo* (Fig. 4). There were 6–8 distinguishable cell layers, starting at the basal cell layer or *stratum basale* formed on the 0.4 μm porous membrane (Fig. 4A). The basal cell layer was composed of cuboidal/columnar shaped cells with large nuclei forming a cell layer with visible desmosomes and/or hemi-desmosomes. The prickle cell layers or *stratum spinosum* (approx. 3–4 cell layers) were comprised of polyhedral-shaped keratinocytes (Fig. 4B) and the *stratum granulosum* (approx. 1–2 cell layers) demonstrated keratohyalin granule-producing cells (identified by white arrows in Fig. 4C). The outer-most 2–3 cell layers showed keratinisation and “pyknosis” (nuclei and organelle condensation; identified by black arrows in Fig. 4D).

### Viability of epithelial cell cultures with multi-species biofilms

**Figure 5:**
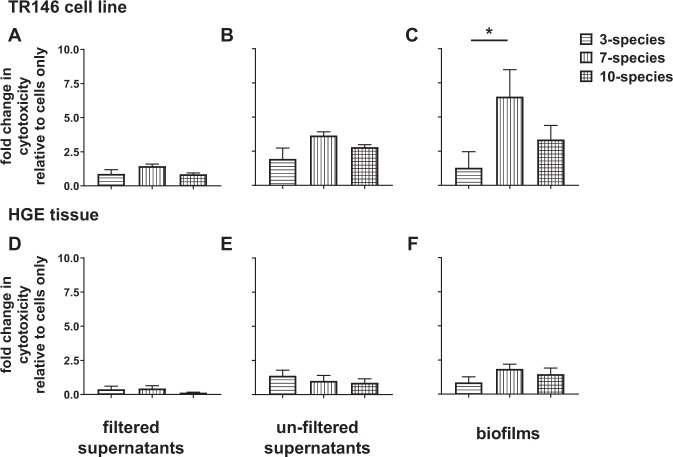
Cytotoxicity of multi-species biofilms on TR146 oral keratinocyte cell line and human gingival epithelium. Oral keratinocytes (grown to 70–80% confluent) (**A–C**) and human gingival epithelium (HGE) (**D–F**) were exposed to filtered and un-filtered biofilm spent growth supernatants, or whole biofilms for 24 hours at 5% CO_2_, 37 °C. Following incubation, keratinocyte or HGE spent media were assessed for the presence of Lactate Dehydrogenase (LDH) as a measure of cell death using the Pierce LDH cytotoxicity assay kit. Data presented as fold change in cytotoxicity relative to cells only minus bacteria, representative of n = 3–4 from one experiment for filtered and un-filtered supernatants, and n = 3–4 from two experiments for whole biofilm co-cultures. Statistical analysis performed using one-way ANOVA with Tukey’s multiple comparison post-test (*p < 0.05).

In order to test the robustness of the three-dimensional epithelial cell model for bacterial co-culture, we assessed the histology and viability of the HGE tissue following exposure to biofilm supernatants and whole biofilms (Fig. 5). Notably, there was no visible change in the histological appearance of the HGE tissue following overnight culture with biofilm supernatants or biofilms (Supplementary Fig. 3). For comparative purposes in viability assessment, a monolayer-forming TR146 keratinocyte cell line was exposed to biofilm supernatants and whole biofilms in a similar manner as HGE tissue. TR146 cells and HGE tissue remained viable following exposure to biofilm supernatants filtered through 0.2 μm membranes, consisting of bacterial products released by the mature biofilm, minus whole bacterial cells (Figs. 5A and D). Following co-culture with un-filtered biofilm supernatants, containing a mixture of bacterial proteins and bacteria shed from the biofilm, TR146 cells and HGE tissue also remained largely viable (Figs. 5B and E). Finally, TR146 cells and HGE tissue were incubated with whole biofilms. Thus, the epithelial cells are subject to bacteria shed from the biofilm, as well as continuous exposure to bacterial products produced by cells in the biofilm. The “gingivitis-associated” 7- species biofilm, was significantly more cytotoxic to the TR146 cell monolayer when compared to the “health-associated” 3- species biofilm (6.50 ± 1.96-fold change in cytotoxicity for 7-species biofilms vs. 1.29 ± 0.48-fold change for 3- species biofilms, *p < 0.05; Fig. 5C). In contrast, the HGE tissue maintained high viability following exposure to all multi-species biofilms, regardless of biofilm composition (Fig. 5F).

### Inflammatory response in epithelial cell cultures with multi-species biofilms

**Figure 6:**
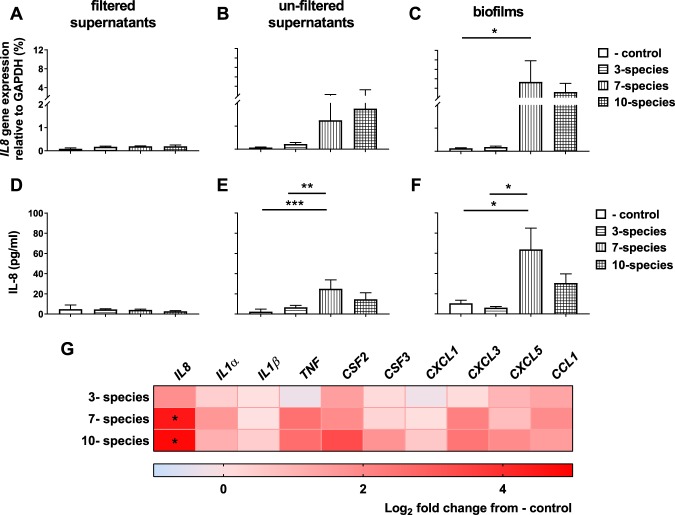
Gene expression profile and IL-8 protein release in human gingival epithelium (HGE) following exposure to oral biofilms. HGE was exposed to filtered and un-filtered biofilm spent growth supernatants, or whole biofilms for 24 hours at 5% CO_2_, 37 °C. Negative control HGE tissue was co-cultured with sterile artificial saliva only. Interleukin-8 gene expression was assessed by quantitative PCR and gene expression calculated relative to the housekeeping gene *GAPDH* (**A–C**). Spent HGE tissue supernatant was assessed for IL-8 protein by ELISA (**D–F**). Panel G shows a heat map depicting the log_2_ fold change in gene expression of pro-inflammatory genes in HGE following exposure to oral biofilms assessed by multiplex qPCR. Data in panels (**A–F**) shown as mean +/− SEM representative of n = 3–4 from one experiment for HGE-biofilm supernatant experiments, and n = 3–4 from two separate experiments for HGE incubation with whole biofilms. Statistical analysis was performed using a one-way ANOVA with Tukey’s multiple comparison post-test (*p < 0.05, **p < 0.01).

The inflammatory response in the HGE tissue was investigated following exposure to biofilm-spent supernatants and multi-species biofilms. Firstly, *IL8* gene expression and IL-8 protein release were assessed in HGE tissue after co-culture with biofilm supernatants. IL-8 is a potent chemoattractant produced by epithelial cells, including gingival tissue, during microbial challenge^7,28,29^. Following exposure to filtered supernatants from the multi-species biofilms, *IL8* gene expression and IL-8 protein release in HGE tissue was comparable to AS only (negative control) (Figs. 6A and 6D). Upon stimulation with un-filtered biofilm spent growth supernatants, there were no significant changes in *IL8* gene expression in HGE tissue, although a trend towards an increase in tissue stimulated with 7- and 10- species biofilm supernatants was observed (Fig. 6B). At a protein level, HGE produced more IL-8 following co-culture with 7- species biofilm spent growth supernatants than 3- species biofilm supernatants and AS only (Fig. 6E; 25.07 ± 3.56 pg/ml for 7- species biofilm supernatants vs. 6.61 ± 0.95 pg/ml for 3- species biofilm supernatants and 2.34 ± 1.47 pg/ml for AS only, **p < 0.01 and ***p < 0.001, respectively).

Following integration of biofilms into the three-dimensional HGE co-culture model, we observed a differential inflammatory response in the HGE tissue following exposure to the “health-associated”, “gingivitis-associated” and “periodontitis-associated” biofilms. Firstly, to assess whether these differential responses were due to differences in bacterial load or composition in the three biofilm co-culture models (as seen in Fig. 3A–C), TR146 epithelial cells were exposed to serially diluted 3-, 7- and 10- species cultures. It may be postulated that an increase in bacterial load in the 7- and 10- species biofilms compared to 3- species biofilms, irrespective of composition, may drive inflammation in epithelial cells *in vitro*. Here, the 7-species suspension induced significantly greater *IL8* expression in TR146 cells compared to 3- species culture at 10^7^, 10^6^ and 10^5^ dilution (Supplementary Fig. 4; *p < 0.05). This suggests that disease-associated organisms such as *Fusobacterium* species (*F. nucleatum* and *F. nucleatum* spp. *vincentii*) present in the 7- species suspensions may be driving an elevated *IL8* inflammatory response in TR146 cells. Conversely, there were no significant changes between 3- and 10- species co-cultures at any serial dilution. In the HGE co-cultures, there was an up-regulation in *IL8* gene expression in 7- species biofilm-stimulated HGE tissue compared to AS only (*p < 0.05), whilst a trend in up-regulation was observed in tissue cultured with 10- species biofilms, compared to 3- species biofilms or AS only, although this difference was not statistically significant (Fig. 6C). At a protein level, IL-8 released by HGE tissue varied in response to the different multi-species biofilms. IL-8 release was significantly higher from HGE tissue co-cultured with 7- species biofilms compared with 3- species biofilms and AS only (63.87 ± 20.99 pg/ml for 7- species biofilm vs. 6.12 ± 1.13 pg/ml for 3- species biofilm and 10.49 ± 2.97 pg/ml for AS only, both *p < 0.05; Fig. 6F). Interestingly, *IL8* gene expression and IL-8 protein release was comparable between 3- species biofilms and AS only, suggesting that oral *Streptococcus*, often termed as commensal bacterial species inhabiting the oral cavity^30^, had little inflammatory effect on the HGE tissue. There were no significant changes in IL-8 release from HGE tissue following exposure to 10- species biofilms compared to other biofilm co-cultures or AS only (p > 0.05), which may possibly be due to *P. gingivalis*-derived gingipains degrading the protein^31^.

Broader investigation of the HGE transcriptional response demonstrated an up-regulation of multiple inflammatory cytokine/chemokine mediators in HGE tissue exposed to 7- and 10- species biofilms (Fig. 6G). *IL8* was the only significantly up-regulated gene in HGE tissue following co-culture with 7- species and 10- species biofilms, compared to AS only (both *p < 0.05). However, there was also an increased expression *TNF* and *CXCL3* in HGE tissue co-cultured with 7- species and 10- species biofilms, although these changes were not significant*. TNF* encodes for TNF-α a pro-inflammatory cytokine involved in acute phase of infection and produced by gingival epithelial cells in response to bacterial stimulation^32,33^. *CXCL3* produces a chemokine thought to have a role in CXCR2 receptor signalling involved in neutrophil and monocyte attraction. Other notable up-regulated genes were *IL1α*, a gene encoding IL-1α, a member of IL1 family of pro-inflammatory cytokines, and *CCL1*, a potent chemoattractant of T cells and monocytes^34^ in HGE tissue co-cultured with 7- species biofilm. The 10-species biofilm induced an up-regulation of *CFS3*, or granulocyte-colony stimulating factor and *CXCL5*, another ligand for CXCR2 receptor involved in monocyte attraction and activation. Generally, the 3- species biofilm had negligible effect on the HGE tissue, with only a subtle up-regulation in gene expression of *IL8*, *CSF2* (granulocyte-macrophage colony-stimulating factor), *CXCL5* and *CCL1* relative to HGE tissue cultured with AS only. Taken together, data presented here suggests that 7- and 10- species biofilms induce a pro-inflammatory response in the HGE tissue, with *IL8* the most up-regulated gene following co-culture with these biofilms.

### Differential inflammatory response in immune cells cultured with HGE tissue

We next sought to integrate immune cells into the HGE three-dimensional co-culture to mimic the *in vitro* interactions between HGE tissue and immune cells. We sought to evaluate both the effect of immune cells on epithelial cells, and vice-versa, as well as the overall culture. To do this, PBMCs and CD14^+^ monocytes were isolated and cultured in maintenance media +/− HGE tissue. *IL8* gene expression in HGE tissue and PBMCs/monocytes was assessed and IL-8 protein detected in media from mono- and co-cultures.

**Figure 7:**
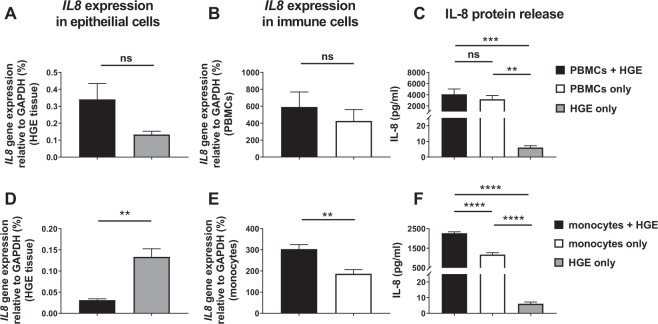
Inflammatory response in immune cells co-cultured with human gingival epithelium. Peripheral blood mononuclear cells (PBMCs) or isolated CD14^+^ monocytes were cultured at a concentration of 1.0 × 10^6^ PBMCs/ml or 0.5 × 10^6^ monocytes/ml with and without human gingival epithelium (HGE) for 24 hours at 5% CO_2_, 37 °C. Interleukin-8 gene expression relative to *GAPDH* was calculated in HGE tissue (**A and D**) and immune cells (**B and E**) using quantitative PCR. Spent supernatant from mono- and co-cultures were assessed for IL-8 protein by ELISA (**C and F**). Data representative of mean +/− SEM representative of n = 3–4 from two separate experiments. Statistical analysis was performed using either an unpaired t test or one-way ANOVA with Tukey’s multiple comparison post-test (**p < 0.01, ***p < 0.001, ****p < 0.0001).

There was no significant difference in *IL8* gene expression in HGE tissue cultured +/− PBMCs (Fig. 7A), nor in PBMCs cultured with or without HGE tissue (Fig. 7B). The concentration of IL-8 in PBMC only cultures and PBMC/HGE co-cultures was significantly higher than HGE-generated IL-8 (4094 ± 942 pg/ml in PBMC co-culture with HGE and 3197 ± 688 pg/ml in PBMC mono-culture vs. 6.124 ± 1.125 pg/ml for HGE only, ***p < 0.001 and **p < 0.01 respectively; Fig. 7C). However, there were no significant differences in the IL-8 concentration in PBMCs only cultures vs. PBMC/HGE co-cultures.

CD14^+^ monocytes were also cultured with and without HGE tissue. *IL8* gene expression in HGE tissue was down-regulated when co-cultured with monocytes (0.1332 ± 0.0194% relative gene expression in HGE tissue minus monocytes vs. 0.0313 ± 0.0029% in HGE plus monocytes, **p < 0.01; Fig. 7D). On the contrary, *IL8* gene expression in monocytes was significantly increased following co-culture with HGE tissue, compared to monocytes cultured in media only (303.4 ± 20.96% relative gene expression in monocytes plus HGE vs. 186.8 ± 19.29% in monocytes minus HGE, **p < 0.01; Fig. 7E). As with PBMCs, IL-8 protein release was elevated in monocyte only cultures, when compared to HGE tissue only (1177 ± 93.05 pg/ml in monocytes in media only vs. 6.124 ± 1.125 pg/ml in HGE mono-culture only, ****p < 0.0001; Fig. 7F). However, unlike the PBMC/HGE co-cultures, the concentration of IL-8 was significantly higher in monocyte/HGE co-cultures, when compared to monocytes only (2267 ± 71.45 pg/ml IL-8 release in monocyte and HGE tissue co-cultures compared with 1177 ± 93.05 pg/ml in monocytes in media only, ****p < 0.0001; Fig. 7F). Therefore, taken together, these data show that HGE tissue induces an up-regulation of *IL8* in monocytes, resulting in increased IL-8 in monocyte/HGE co-cultures.

**Figure 8:**
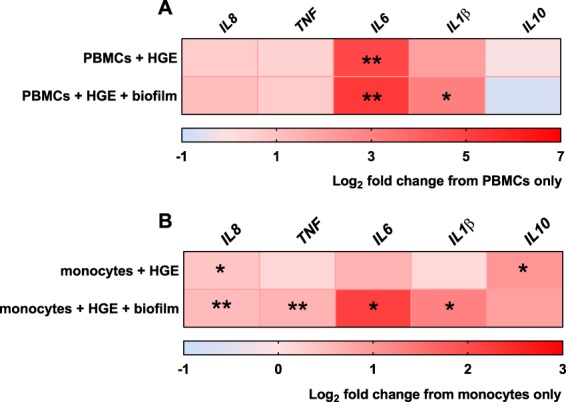
Inflammatory response in immune cells co-cultured with biofilm-stimulated human gingival epithelium. Peripheral blood mononuclear cells (PBMCs) or isolated CD14^+^ monocytes were cultured alone, with human gingival epithelium (HGE) or 7- species biofilm-stimulated HGE for 24 hours at 5% CO_2_, 37 °C. Heat map data shows the log_2_ fold change in gene expression of pro-inflammatory genes (*IL8*, *TNF*, *IL6*, *IL1β*) and *IL10* in PBMCs or CD14^+^ monocytes following incubation with HGE + /− 7 species biofilm, relative to immune cells only. Heat map data displayed as mean value representative of n = 3 from one experiment. Statistical analysis was performed using one-way ANOVA with Tukey’s multiple comparison post-test (*p < 0.05, **p < 0.01).

To further mimic the interactions between HGE tissue and immune cells in oral inflammation, 7- species biofilms were introduced into the HGE tissue/immune cell co-cultures. The 7- species biofilm was selected as stimulant as it appeared to elicit the greatest pro-inflammatory response in the HGE tissue (as seen in Fig. 6). Firstly, it is noteworthy that no culturable bacteria were detected in the media underneath the tissue during the duration of the experiments, confirming that viable bacteria had not disseminated through the tissue and directly stimulated immune cells. Figure 8A shows the fold change in PBMC gene expression of *IL8*, *TNF*, *IL6*, *IL1β* and *IL10* following co-culture with HGE tissue + /− 7- species biofilm, relative to PBMCs only. After incubation with HGE, there was an up-regulation in gene expression of *IL6* in PBMC/HGE co-cultures, irrespective of biofilm stimulation (both **p < 0.01) and *IL1β*, in PBMCs incubated with HGE stimulated with 7- species biofilm (**p < 0.01). There was no change in gene expression of *IL8*, *TNF* or *IL10* in PBMCs following culture with HGE +/− biofilm, compared to PBMCs only. There were also no changes in expression of any gene in PBMCs cultured with HGE vs. biofilm-stimulated HGE (Supplementary Fig. 5).

There were more significant changes in gene expression in monocytes cultured with HGE + /− 7- species biofilm (Fig. 8B; % expression shown in Supplementary Fig. 6). In addition to *IL8* (Fig. 7E and Supplementary Fig. 6A), *IL10* was also significantly up-regulated in monocytes cultured with HGE tissue compared to monocytes cultured alone (Supplementary Fig. 6E; *p < 0.05). Conversely, pro-inflammatory genes *IL8*, *TNF*, *IL6* and *IL1β* were all significantly up-regulated in monocytes cultured with HGE tissue stimulated by 7- species biofilm, compared to monocytes cultured alone (*IL8*, *TNF* both **p < 0.01; *IL6*, *IL1β* both *p < 0.05, respectively). Two genes were differentially expressed in monocytes cultured with biofilm-stimulated HGE vs. HGE only minus biofilm stimulation; *TNF* (Supplementary Fig. 6B; **p < 0.01) and *IL6* (Supplementary Fig. 6C; *p < 0.05) were both significantly up-regulated in monocytes cultured with biofilm-stimulated HGE. Taken together results from the preceding section show that HGE tissue can orchestrate the gene expression profile of immune cells in the media underneath possibly via the production of soluble mediators such as IL-8, an effect that is more pronounced following stimulation of HGE with 7- species biofilm.

## Discussion

Oral biofilm-host cell co-culture models are important tools in understanding the pathogenesis of oral inflammatory diseases. Work from the present study has shown that the multi-species biofilms containing “health-associated”, “gingivitis-associated” and “periodontitis-associated” microorganisms exhibit unique morphological features as assessed by different microscopic techniques. The HGE tissue used throughout this study formed a 6–8 cell-layered epithelium characteristic of gingivae *in vivo* and maintained a high level of viability following exposure to multi-species biofilms, unlike our epithelial cell line monolayer. HGE exhibited a unique cytokine gene and protein expression profile after co-culture with the multi-species biofilms, suggesting the composition and complexity of these biofilms can influence the inflammatory response in HGE tissue. In addition, we observed a differential inflammatory response in immune cells co-cultured with and without HGE tissue, and in biofilm-stimulated HGE co-cultures. In this model, HGE controls the inflammatory state of immune cells potentially through the production of tissue-derived soluble mediators, in a manner that partially mimics the interactions between epithelial cells and tissue-resident immune cells *in vivo*.

The majority of previous studies seeking to assess the inflammatory response in oral epithelial cells have used primary or immortalised cell lines that form monolayers following culture. Monolayer cell models are cheaper, easy to manipulate and highly reproducible for the study of host-bacteria interactions. However, *in vivo*, the oral mucosa surrounding the tooth consists of largely multi-layered keratinised gingival epithelium and non-keratinised sulcular and junctional epithelium. Here, we show that “gingivitis-associated” 7- species biofilms induced cell death in a TR146 keratinocyte monolayer, whilst HGE tissue maintained high viability following exposure to all multi-species biofilms. These observations support previous findings describing cell death in epithelial cell monolayers following co-culture with multi-species oral biofilms^8,35^. For example, the work of Guggenheim *et al*. (2009) used a similar co-culture system as the one described in this study, demonstrating that a 9- species subgingival *in vitro* biofilm induces apoptosis in human gingival epithelial cells^35^. Conversely, proteomic studies of similar 3D co-culture systems have observed networks associated with the organization of cytoskeletal intermediate and actin filaments, cell junctions and other proteins involved in cell integrity were altered in response to diseased biofilms^17,36^. However, others showed that a multi-layered oral epithelium remains largely viable after incubation with an oral biofilm containing the “Red complex” consortium of periodontal pathogens^7^. Similar differences in cytotoxicity were observed using other *in vitro* two-dimensional and three-dimensional cell models. Studies of the effects of dental resins on two-dimensional and three-dimensional gingival cell cultures demonstrate significant increases in cell death in monolayers following treatment compared with a multi-layered cell model^37^. Furthermore, two-dimensional skin cell monolayers are more susceptible to cytotoxic agents than three-dimensional grown counterparts^38^. It is apparent that the most important defensive feature of gingival epithelium is its thick, multi-layered phenotype, providing the first line of cellular protection to the external environment. It would be of interest to study whether non-keratinised oral epithelium is equally protected from biofilm-mediated cytotoxicity, or if the keratinised layer is functionally important in this protection. Furthermore, one limitation of this study was that biofilms were not in direct contact with HGE. Previous studies have reported that *P. gingivalis*, present in our “periodontitis-associated” biofilm model, is able to be internalised by and survive within gingival epithelial cells^39–41^. Likewise, ensuing research has found that this organism is capable of bypassing the epithelial barrier in a 3D gingival epithelial and fibroblast spheroid model to induce an inflammatory response^42^. Thus, future work would benefit from assessing whether these contact-dependent mechanisms can be mimicked in this model.

Oral epithelial cells display differential inflammatory responses following culture with mono- and multi-species biofilms of varying compositions in a species-specific manner^5–8^. The works of Peyyala *et al*. (2012 and 2013) demonstrated a clear hierarchy in the bacterial induction of inflammatory response in oral epithelial cells; mature biofilms containing pathogenic bacteria were more pro-inflammatory than biofilms comprising of commensal microorganisms, whilst mono-species biofilms and planktonic bacteria were less pro-inflammatory^5,6^. Interestingly we observed a similar phenomenon in this study using TR146-bacterial suspension and HGE-biofilm co-cultures. In the TR146 co-cultures, the 7- species suspension induced the greatest increase in *IL8* gene expression in epithelial cells whilst the 3- species culture induced the lowest response of all three co-cultures at each dilution. This was indicative that the composition of the 7-species biofilm, as opposed to bacterial load, was dictating the inflammatory response within co-cultured epithelial cells. In the HGE tissue co-cultures, the “health-associated” 3- species biofilm had a negligible effect on the inflammatory response in the HGE in regard to both gene and protein expression. In contrast, the 7- and 10- species biofilms, comprised of microorganisms associated with gingival inflammation such as *F. nucleatum*, *A. actinomycecomitans* and *P. gingivalis*, induced a pro-inflammatory gene response in the HGE tissue following co-culture. *IL8* gene expression was the most upregulated gene in HGE tissue stimulated by 7- species and 10- species biofilms. At the protein level, we observed an increase in IL-8 cytokine release in the spent tissue supernatant of HGE co-cultured with 7- species biofilms. Similar findings have been described elsewhere, with IL-8 production by epithelium a primary response to biofilms containing *A. naeslundii* and *F. nucleatum*^5,6^. Interestingly, although at the gene level *IL8* was upregulated, IL-8 protein release from HGE was not significantly different than the control following 10- species biofilm challenge. These observations are in line to those described by Belibasakis and colleagues (2013), whereby “Red complex” microorganisms may degrade host proteins^7^. Indeed, it is well established that *P. gingivalis* can degrade IL-8 to modulate the immune response^31,43^. We therefore propose that similar mechanisms are in place in the tissue and 10- species biofilm co-culture.

In addition to the well-documented pathogenicity of the microorganisms, this elevated pro-inflammatory response may also be due to the complex nature of the mature “diseased” biofilms; our findings correspond with previous studies that mature biofilms are more pro-inflammatory than less‐complex biofilms^44^. Such bacteria-host interactions are driven by pattern recognition receptors (PRRs) associated with cellular signalling pathways including mitogen-activated protein kinase (MAPK), protease-activated receptors (PAR) and Toll-like receptors (TLR)^45,46^. Secreted bacterial products such as lipopolysaccharides (LPS) and proteases (e.g., gingipains in *P. gingivalis*) also known as pathogen-associated molecular patterns (PAMPs), can orchestrate the immune response in oral epithelial cells via these cell signalling pathways^47–51^. Interestingly, there is now a plethora of research evidence that commensal and pathogenic bacteria possess unique LPS structures that differentially modulate signalling cascades in epithelial and immune cells^46,52–54^. It is feasible that similar mechanisms are present in our 3D model whereby biofilms associated with disease induce pro-inflammatory responses via PRRs in HGE whilst “health-associated” biofilms are more immunomodulatory and are therefore unable to activate similar pathways. Future studies assessing the expression of PRRs in HGE tissue pre- and post-stimulation with biofilms associated with health and disease may begin to clarify these mechanisms.

The oral epithelial cell response to a microbial challenge is an essential “preliminary warning signal” in controlling the innate and adaptive immune responses. Thus, to study the effects of HGE responses on immune cells, we incorporated PBMCs and purified monocytes into our three-dimensional co-culture model to mimic cell-cell communication between epithelial cells and immune cells. PBMCs were initially chosen for co-culture as these contain populations of lymphocytes such as T cells (70–85%), B cells (5–10%), NK cells (5–20%) and innate lymphoid cells (<0.1%), as well as mononuclear phagocytes such as monocytes (10–20%) and dendritic cells (1–2%)^55^, each of which have been identified within gingival tissue^10^. For comparison, we isolated PBMC-derived CD14^+^ monocytes for HGE co-culture. Monocytes were chosen as the role of recruited monocytes and tissue resident macrophages in oral health and disease remains to be fully elucidated, although there are some studies that have identified monocytes/macrophages in gingival tissue biopsies. The number of CD14^+^, CD16^+^ monocytes/macrophages were increased in gingival biopsies of patients with chronic periodontitis^56^, whilst there is also evidence for an increase in M1 (pro-inflammatory state) macrophages in inflamed gingivae^57^. However, the mechanisms by which these cells infiltrate the tissue during inflammation are not fully understood. *In vitro* studies like the one described here may begin to allude to potential mechanisms.

We describe unique cytokine gene and protein expression profiles in PBMCs and monocytes cultured with and without HGE tissue in our three-dimensional model. There was a significant up-regulation of *IL8* gene expression in monocytes incubated with HGE tissue than without, meaning that HGE tissue controls gene expression in isolated monocyte cultures via production of soluble mediators, likely IL-8. As IL-8 is an autocrine chemokine^58^, we propose that HGE- and monocyte-derived IL-8 is stimulating further production of IL-8 by the monocytes via the CXCR1 and CXCR2 receptors, an effect that is lost following incubation of PBMCs with HGE. This may be explained by the different cell populations within the PBMC co-culture compared to the purified monocyte co-culture; other cell types within the PBMC population such as T regulatory cells have been shown to be drive M2 differentiation in monocyte populations, suppressing pro-inflammatory responses such as IL1-β, TNF-α, IL-6 and IL-8 production^59,60^. Future work may merit consideration of whether co-culture of HGE tissue induces polarization of monocytes (or monocytes in PBMC populations) into M1 or M2 macrophages. Nonetheless, for both PBMCs and monocytes we observe greater changes in gene expression following culture with biofilm-stimulated HGE tissue, which suggests that the “inflammatory state” of the tissue can impact the responses in co-cultured immune cells *in vitro*. We hypothesise that the mechanisms we have observed *in vitro* are likely present *in vivo*. In a “resting state”, cell-cell communication between the epithelium and immune cells is critical in regulating the inflammatory response and maintaining tissue homeostasis. Conversely, following perturbation of the epithelium, rapid cell-cell signalling is key for removal of any pathogenic threat^61^. Unfortunately, we were unable to assess the ability of the otherwise chemoattractant IL-8 in inducing migration of cells through the tissue due to the 0.4 µm pore size of the insert membranes. Other work using similar two-dimensional and three-dimensional epithelial models, describe the use of inverted culture inserts or trans-well systems to measure migration of neutrophils through epithelial cells^62–64^. Future work could benefit from the integration of neutrophils or monocytes into an inverted version of our model. Using similar oral tissue grown on larger porous membranes, within this inverted model, would allow us to assess the cell chemotaxis through the epithelium following stimulation with biofilms.

In summary, we have described the development of a three-dimensional gingivae biofilm co-culture model to study the inflammatory responses in epithelium following exposure to multi-species oral biofilms. In addition, to our knowledge, this is the first publication to describe a three-dimensional gingivae-biofilm co-culture model with integration of immune cells for investigating biofilm-epithelium-immune cell interactions. Using *in vitro* models as described in this study may help dissect the cell-cell communication pathways that exist between oral biofilms, epithelial cells and immune cells *in vivo*, and how such interactions may drive the onset of oral inflammatory diseases such as gingivitis and periodontitis.

## Supplementary information

supplementary data file
