# Supplementary material for: Biofilm-stimulated epithelium modulates the inflammatory responses in co-cultured immune cells
Source: Sci Rep. 2019 Oct 31;9:15779. doi: 10.1038/s41598-019-52115-7 (PMC6823452; doi:10.1038/s41598-019-52115-7)
Supplement: Supplementary file 1 — supplementary data file [file 41598_2019_52115_MOESM1_ESM.docx]

**Biofilm-stimulated epithelium modulates the inflammatory responses in co-cultured immune cells.**

**Jason L Brown, William Johnston, Chris Delaney, Ranjith Rajendran, John Butcher, Shaz Khan, David Bradshaw, Gordon Ramage, Shauna Culshaw**

**Supplementary Figures**


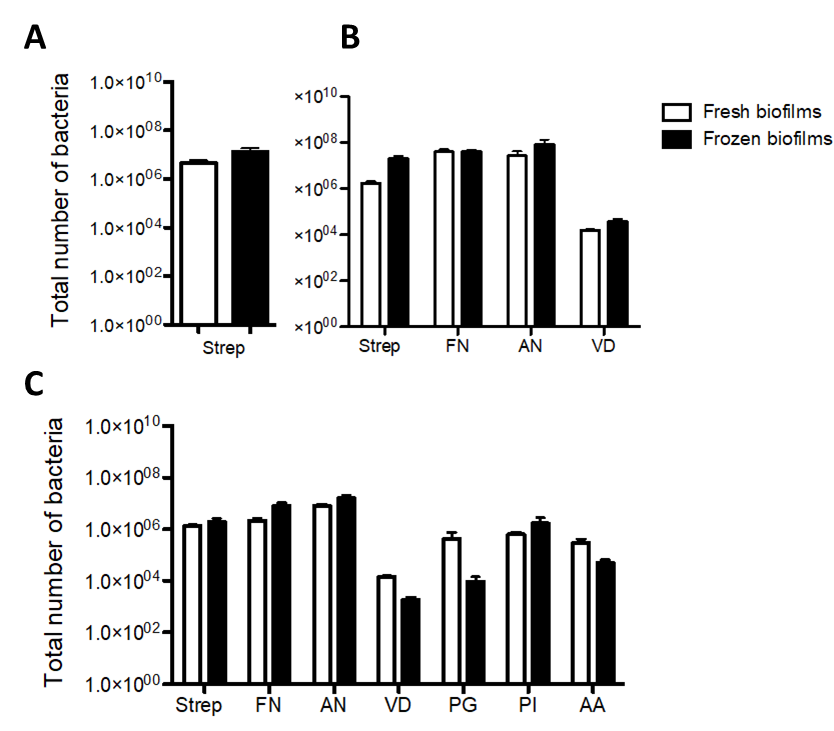


**Supplementary Figure 1 – Comparison of fresh and frozen biofilm composition for the 3-, 7- and 10- species biofilms.** Mature 3-species (A), 7-species (B) and 10-species (C) biofilms were grown and DNA extracted immediately (fresh; white bars) or following revival from -80^o^C (frozen; black bars). The biofilms were sonicated to detach the biofilm from the coverslip and bacterial DNA was extracted using the QIAamp DNA Mini Kit. The proportion of each species in final composition was quantified using SYBR® GreenER based quantitative PCR. Data shown are mean ±SEM of n=3 from 3 independent experiments. No statistical differences were observed as assessed using two-tailed Student’s t-test for each microorganism. Abbreviations; Strep = *Streptococcus* species, FN = *Fusobacterium* species, AN = *A. naeslundii*, VD = *V. dispar*, PG = *P. gingivalis*, PI = *P. intermedia*, AA = *A. actinomycetemcomitans*.





**Supplementary Figure 2 – Standard curves used for all 10 bacterial species**. Mono-cultures of all bacteria were cultured overnight under appropriate conditions then standardized to 1 x 10^8^ CFU/ml. DNA was extracted using the QIAamp DNA Mini Kit then serially diluted to generate a standard curve from 1 x 10^8^ – 1 x 10^3^. DNA was quantified using SYBR GreenER based qPCR and bacterial specific primer sets. Data was compiled by plotting raw Ct values against CFU/ml and R^2^ determined using a linear regression analysis. Unknown bacterial counts from multi-species biofilms could then be quantified by extrapolating from the appropriate curve. Data shown are mean ± SEM of DNA extracted from mono-cultures from two independent experiments.


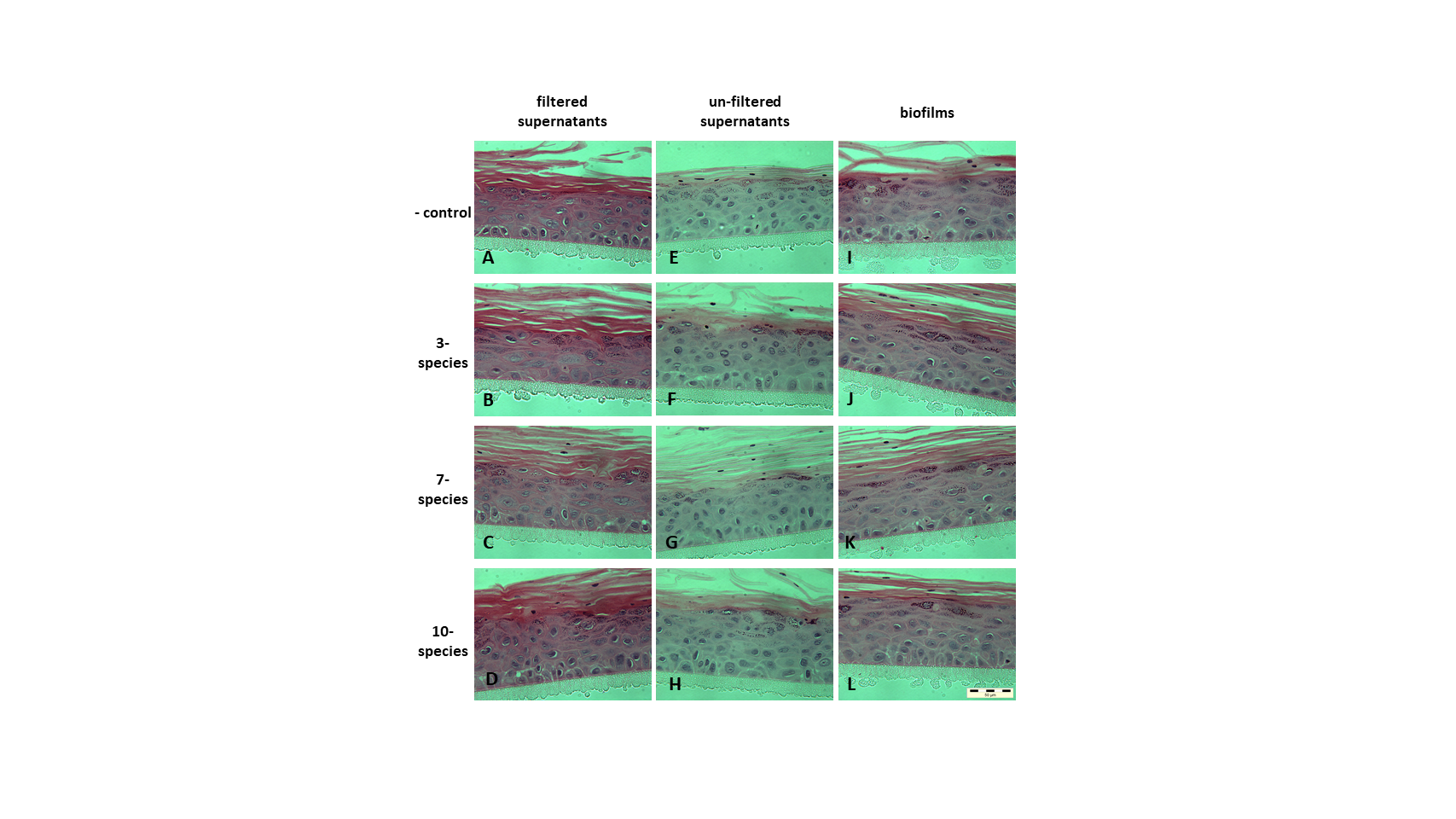


**Supplementary Figure 3 – Histological analysis of human gingival epithelium (HGE) following co-culture with biofilm supernatants or mature biofilms.** Haematoxylin and Eosin stained histological sections of HGE tissue following exposure to final day biofilm spent growth supernatants in artificial saliva (AS) filtered through 0.2 μm filters (A-D), un-filtered supernatants (E-H) or fully mature oral biofilms (I-L). The – control tissue was cultured with sterile AS only. Histological images representative of one tissue section from one field of view per experiment.


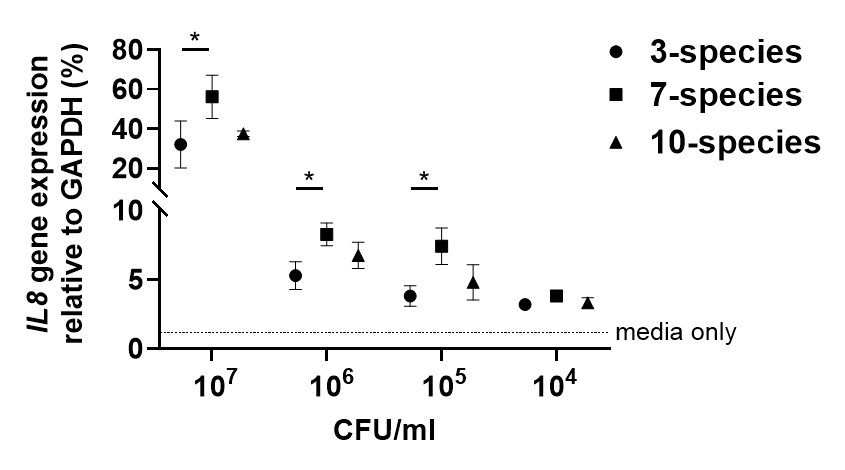


**Supplementary Figure 4 – TR146 cell response to multi-species bacterial suspensions.** All microorganisms were standardised to the final CFE/ml representative of the composition of fully mature 3-, 7- and 10- species biofilms (as shown in Figure 3A-C). These were serially diluted from 10^7^ to 10^4^ prior to exposure to confluent TR146 cell monolayers in 24-well plates then incubated at 37^o^C, 5% CO_2_ for 24 hours. Following incubation, *IL8* gene expression in TR146 cells was assessed by quantitative PCR. Data shown are mean ±SEM of n=3 and statistical analysis was performed using a one-way ANOVA with Tukey’s multiple comparison post-test (* p < 0.05).





**Supplementary Figure 5 – Gene expression in peripheral blood mononuclear cells (PBMCs) following culture with human gingival epithelium.** PBMCs were isolated from peripheral blood and resuspended to 1 x 10^6^ cells/ml. PBMCs were cultured alone (white bars), with HGE (black bars), or with 7- species biofilm-stimulated HGE (striped bars) for 24 hours at 5 % CO_2_, 37^o^C. Gene expression for all genes were assessed by quantitative PCR and gene expression calculated relative to the housekeeping gene *GAPDH*. Data shown as mean +/- SEM representative of n=3 from one experiment. Statistical analysis was performed using a one-way ANOVA with Tukey’s multiple comparison post-test (* p < 0.05, ** p < 0.01).

**

**

**Supplementary Figure 6 – Gene expression in CD14^+^ monocytes following culture with human gingival epithelium.** CD14^+^ monocytes were purified from PBMC populations and resuspended to 0.5 x 10^6^ cells/ml. CD14^+^ monocytes were cultured alone (white bars), with HGE (black bars), or with 7- species biofilm-stimulated HGE (striped bars) for 24 hours at 5 % CO_2_, 37^o^C. Gene expression was assessed by quantitative PCR and gene expression calculated relative to the housekeeping gene *GAPDH*. Data shown as mean +/- SEM representative of n=3 from one experiment. Statistical analysis was performed using a one-way ANOVA with Tukey’s multiple comparison post-test (* p < 0.05, ** p < 0.01).
